# A Novel SERS Silent-Region Signal Amplification Strategy for Ultrasensitive Detection of Cu^2+^

**DOI:** 10.3390/molecules30102188

**Published:** 2025-05-16

**Authors:** Jiabin Su, Kaixin Chen, Ping Zhou, Nan Li

**Affiliations:** Key Laboratory of Biomaterials of Guangdong Higher Education Institutes, Department of Biomedical Engineering, Jinan University, Guangzhou 510632, China; q82304555@stu2022.jnu.edu.cn (J.S.); kaychan10095@outlook.com (K.C.); tmyield@jnu.edu.cn (P.Z.)

**Keywords:** surface-enhanced Raman scattering, signal amplification strategy, silent region detection, cyanide functionalization, Cu^2+^ detection

## Abstract

Due to its unique molecular fingerprinting capability and multiplex detection advantages, surface-enhanced Raman scattering (SERS) has shown great application potential in the field of biological analysis. However, the weak signal intensity and large background interference significantly limited the application of SERS in biosensing and bioimaging. Loading a large amount of Raman molecules with signal in the silent region on the hotspots of the electromagnetic field of the SERS substrate can effectively avoid severe background noise signals and significantly improve the signal intensity, making the sensitivity and specificity of SERS detection remarkably improved. To achieve this goal, a new SERS signal-amplification strategy is herein reported for background-free detection of Cu^2+^ by using Raman-silent probes loaded on cabbage-like gold microparticles (AuMPs) with high enhancement capabilities and single-particle detection feasibility. In this work, carboxyl-modified AuMPs were used to enable Cu^2+^ adsorption via electrostatic interactions, followed by ferricyanide coordination with Cu^2+^ to introduce cyano groups, therefore generating a stable SERS signal with nearly zero background signals owing to the Raman-silent fingerprint of cyano at 2137 cm^−1^. Based on the signal intensity of cyano groups correlated with Cu^2+^ concentration resulting from the specific coordination between Cu^2+^ and cyanide, a novel SERS method for Cu^2+^ detection with high sensitivity and selectivity is proposed. It is noted that benefiting from per ferricyanide possessing six cyano groups, the established method with the advantage of signal amplification can significantly enhance the sensing sensitivity beyond conventional approaches. Experimental results demonstrated this SERS sensor possesses significant merits towards the determination of Cu^2+^ in terms of high selectivity, broad linear range from 1 nM to 1 mM, and low limit of detection (0.1 nM) superior to other reported colorimetric, fluorescence, and electrochemical methods. Moreover, algorithm data processing for optimization of SERS original data was further used to improve the SERS signal reliability. As the proof-of-concept demonstrations, this work paves the way for improving SERS sensing capability through the silent-range fingerprint and signal amplification strategy, and reveals SERS as an effective tool for trace detection in complex biological and environmental matrices.

## 1. Introduction

Surface-enhanced Raman Scattering (SERS) has emerged as a powerful analytical technique [1]. As a vibrational spectroscopy method, SERS uniquely attains single-molecule-level sensitivity, enabling the deciphering of complex molecular fingerprints for direct target analyte identification [2,3,4,5]. Its non-destructive and rapid nature, along with the elimination of the need for sample pretreatment, makes it an indispensable tool for analyzing aqueous biological samples [6].

However, SERS is plagued by several limitations. For example, nanomaterials used in SERS often have uncontrollable size, shape, and irreproducible nanogaps, leading to unstable fluctuations in SERS signal intensity and peak positions [7,8,9,10]. Despite efforts to enhance Raman signal intensity using nanoarrays, achieving the requisite intensity for complex detection remains arduous [11,12,13,14,15]. On the other hand, the fingerprint characteristic peaks of biological molecules and chemical groups in the Raman fingerprint region cause an unavoidable interference [16,17,18,19,20,21]. To overcome these challenges, loading a large amount of Raman molecules with signal in the silent region on the SERS substrate used for signal-particle detection can effectively avoid severe background noise signals and significantly improve the signal intensity. Thus, the development of an effective strategy for SERS detection in the Raman silent-region from 1800 to 2800 cm^−1^, has become a focal point of research in SERS biosensing [20,22,23,24,25,26]. While the previous studies mainly focus on the SERS bioimaging using Raman molecules or nanoprobes with silent-region signal [19,27,28], the exploration of new methods applicable for background-free SERS biosensing to effectively avoid severe background noise signals and significantly improve the signal intensity remains challenging.

To achieve the goal of a significant increase in the sensitivity and specificity of SERS detection, in our previous studies, a SERS method was developed for sensitive detection of hydrogen peroxide (H_2_O_2_) with an ultralow detection limit (1 × 10^−10^ mol/L), using a Raman-silent strategy that 4-mercaptophenylboronic acid (4-MPBA) as an H_2_O_2_-responsive Raman reporter was reacted with H_2_O_2_ converted to a phenol group, which subsequently react with 4-diazonium-phenylalkyne (4-DP), an alkyne-carrying molecule to produce an intense and sharp SERS signal in the Raman-silent region [23]. In addition to that, we have also developed an ultrasensitive SERS sensor to determine cellular oxidative stress based on the H_2_O_2_-induced enzymatic amplification and silent-range Raman fingerprint [29]. On the basis of these works, a deeper investigation on the combination of silent-range Raman fingerprint with signal amplification is strongly desired to make the sensitivity and specificity of SERS detection remarkably improved through introducing a lot of Raman molecules with an intrinsic silent-range Raman fingerprint on the hotspots of the electromagnetic field on the SERS substrate [30,31,32].

As a proof of concept, we herein proposed a new SERS signal-amplification strategy by using cabbage-like gold microparticles (AuMPs) with superior enhancement capabilities and single-particle detection feasibility. Carboxyl-modified AuMPs were used to enable Cu^2+^ adsorption via electrostatic interactions, followed by ferricyanide coordination with Cu^2+^ to introduce cyano groups, generating a stable SERS signal with nearly zero background signals owing to the Raman-silent fingerprint of cyano at 2137 cm^−1^. Based on the signal intensity of cyano groups correlated with Cu^2+^ concentration resulting from the specific coordination between Cu^2+^ and cyanide, a novel SERS method for Cu^2+^ detection with high sensitivity and selectivity is developed. Copper, widely distributed in nature, is an essential trace element for living organisms. Cu^2+^ plays critical physiological roles, including enzymatic catalysis, maintenance of nervous system health, and regulation of hematopoietic functions [33,34,35,36,37]. Thus, the physiological imbalances in Cu^2+^ levels can lead to severe diseases such as liver and kidney damage [38,39] and Alzheimer’s disease [40]. Therefore, accurate detection of Cu^2+^ concentrations in serum are important for clinical diagnosis. Current methods for Cu^2+^ detection include colorimetry [41,42], fluorescence imaging [43,44], electrochemical techniques [45], and surface plasmon resonance (SPR) [46]. However, they often suffer from the disadvantage of operational complexity, insufficient sensitivity, and poor specificity, etc. It is worth noting that, benefiting from per ferricyanide possessing six cyano groups, the established method achieves the advantage of signal amplification, which can significantly enhance the sensing sensitivity superior that of conventional approaches. Moreover, an algorithm for data processing for optimization of SERS original data was further used to improve the SERS signal reliability. This work paves the way for improving SERS sensing capability through the silent-range fingerprint and signal amplification strategy, and reveals SERS as an effective tool for trace detection.

## 2. Results and Discussion

### 2.1. Characterization of SERS Probe

**Figure 1:**
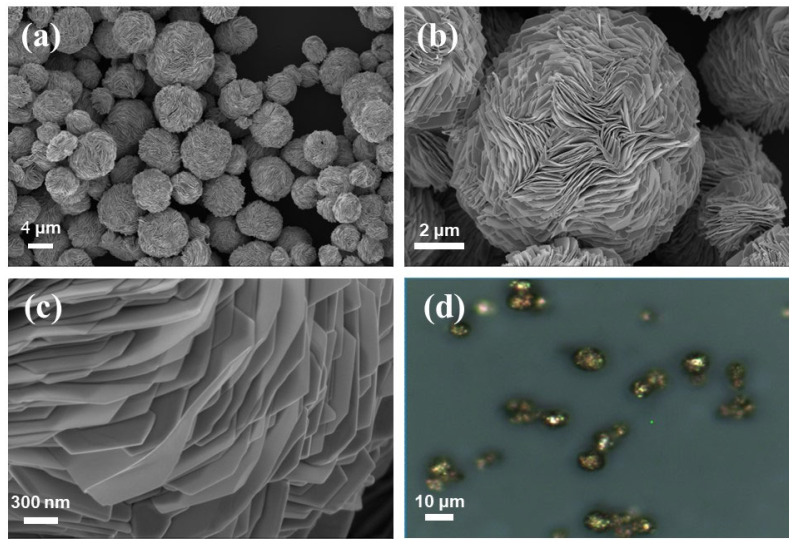
(**a**) Representative SEM images of AuMPs, (**b**) a single particle, (**c**) and its local features. (**d**) The optical microscope image of isolated AuMPs.

We present a “one-pot” synthetic strategy for the preparation of AuMPs as excellent SERS substrates according to the modified previous methodology [47]. The SEM characterization illustrated that spherical AuMPs exhibit densely-packed layer structures with an average diameter of approximately 7 ± 0.92 μm (Figure 1a). The individual AuMPs showed a densely packed layered structure of Au microplates (Figure 1b), providing a large number of sharp edges, sharp corners, as well as nanogaps among different layers (Figure 1c), which can generate hotspots for electromagnetic enhancement (EM). These hotspots play a crucial role in generating intense SERS signals. Figure 1d shows the microscopic image of AuMPs deposited on a silicon wafer. The single bright dot indicates the isolated AuMPs. Due to the diameter of AuMPs being much larger than the size of the laser beam of 2 μm used for the SERS measurement, allowing the measurement of SERS signals on an individual AuMP.

### 2.2. Sensing Mechanism

**Scheme 1:**
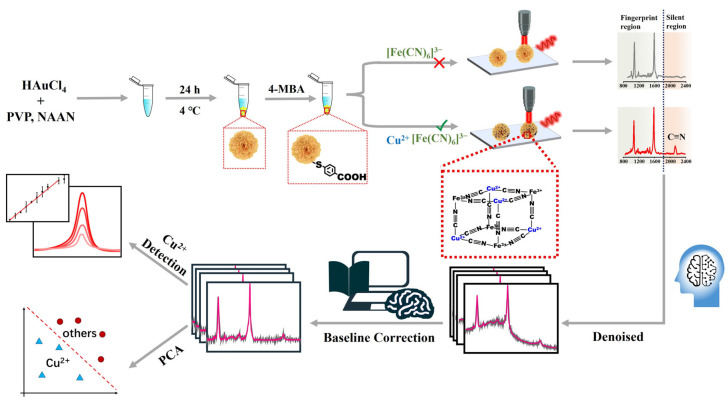
Schematic diagram of the Cu^2+^ detection method based on 4-MBA@AuMP probe using SERS.

In this work, a new SERS signal-amplification strategy is developed for the ultrasensitive detection of Cu^2+^ concentration in the Raman-silent region. Cabbage-like AuMPs with superior enhancement capabilities and single-particle detection feasibility were used as the SERS substrate. The surface of these AuMPs was functionalized with 4-mercaptobenzoic acid (4-MBA) molecules, forming robust Au-S bonds. Subsequently, these 4-MBA-coated AuMPs acted as efficient chelators, capturing Cu^2+^ through electrostatic attractions [48]. The captured Cu^2+^ further underwent coordination with ferricyanide ions, culminating in the formation of a stable Prussian blue-like structure [49]. The distinctive Raman fingerprint of the cyanide moiety, positioned at 2137 cm^−1^ within the characteristic “silent region”, played a crucial role in this detection methodology. Based on the signal intensity of cyano groups correlated with Cu^2+^ concentration resulting from the specific coordination between Cu^2+^ and cyanide, a novel SERS method for Cu^2+^ detection with high sensitivity and selectivity is proposed. It is worth noting that, benefiting from per ferricyanide possessing six cyano groups, the established method with the advantage of signal amplification can significantly enhance the sensing sensitivity beyond conventional approaches. Furthermore, to enhance the precision and reliability of these measurements, advanced algorithms were applied to optimize the acquired signals. The noise reduction algorithm combining baseline correction and Gaussian smoothing is further used for optimization of original data to remarkably improve the SERS signal reliability and enhance the sensing sensitivity (Scheme 1).

### 2.3. Feasibility Demonstration

**Figure 2:**
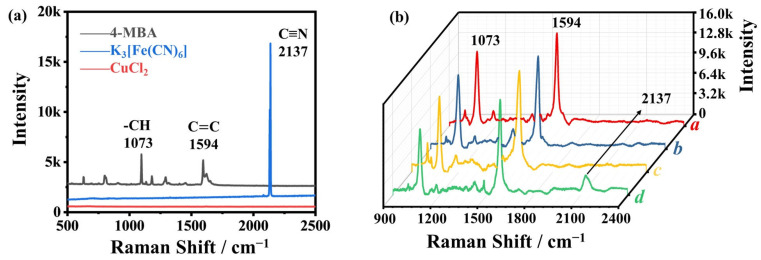
(**a**) The Raman spectra of 4-MBA powder (black line), CuCl_2_ powder (red line), K_3_[Fe(CN)_6_] powder (blue line). (**b**) The Raman spectrum of AuMP@4-MBA (line a), probe+CN (line b), probe@Cu^2+^ (line c), probe@Cu^2+^ @CN (line d).

In the field of Raman spectroscopy (Figure 2a), the characteristic fingerprint spectra of key substances 4-MBA, K_3_Fe(CN)_6_, and CuCl_2_ provide profound insights. The benzene-ring vibrations of 4-MBA display characteristic peaks at 1073 and 1594 cm^−1^ (black line), while the presence of -C≡N is indicated by the peak at 2137 cm^−1^ (blue line). Notably, CuCl_2_ has no discernible peaks within the spectral range of 500–2500 cm^−1^ (red line), highlighting the need for subtle molecular discrimination in complex samples. When 4-MBA self-assembles on AuMPs, distinct Raman peaks appear at specific wavenumbers (1073 and 1594 cm^−1^, line a, Figure 2b). These prominent peaks signify a remarkable enhancement in SERS signals, with the corresponding enhancement factor (EF) calculated to be 1.39 × 10^6^ using established methods [47]. This enhancement exceeds the performance of previously reported SERS substrates, thereby establishing the exceptional sensitivity and specificity of the AuMP@4-MBA platform. Notably, when attempting to combine K_3_[Fe(CN)_6_] with AuMP@4-MBA, the crucial peak at 2137 cm^−1^ does not appear. This absence indicates electrostatic repulsion and the inability to achieve the desired molecular interaction. This selective non-appearance highlights the precise molecular-recognition capabilities of the AuMP@4-MBA system, strengthening its potential for detailed molecular analyses. In a critical series of steps, the AuMP@4-MBA probe was incubated sequentially with Cu^2+^ and K_3_[Fe(CN)_6_], resulting in the emergence of the significant Raman band at 2137 cm^−1^ (line d, Figure 2b). This process is detailed as follows: initially, the probe showed sharp peaks at 1073 and 1594 cm^−1^ (line a), indicating the strong attachment of 4-MBA to AuMPs. Subsequent incubation with ferricyanide (line b) or Cu^2+^ (line c) did not change the probe’s fundamental characteristics. However, a notable change occurred when ferricyanide was added after Cu^2+^ capture, leading to the distinct appearance of a 2137 cm^−1^ peak in the silent region. This specific peak indicated that ferricyanide chelation occurred only after Cu^2+^ binding, emphasizing the probe’s precision and selectivity in molecular recognition. These silent-region peaks not only served as a shield against background interferences but also ensured enhanced sensitivity and confidence in detecting trace biomarkers. Such precision is invaluable, highlighting the probe’s importance in advanced analytical techniques. Further optimization of the capture probes has enhanced the probe’s effectiveness, solidifying its role in cutting-edge analytical methodologies.

**Figure 3:**
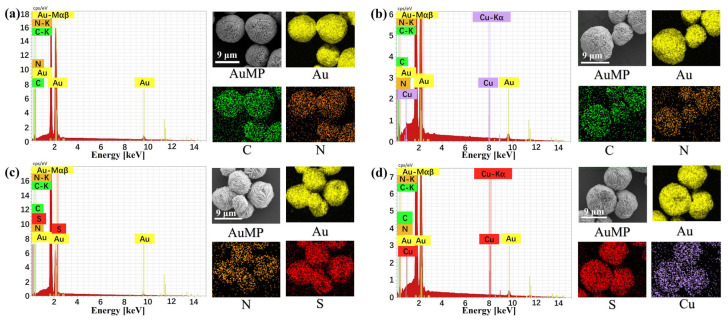
FESEM images of AuMPs and EDX elemental mapping: (**a**) AuMPs, (**b**) AuMP+Cu^2+^, (**c**) AuMP@4-MBA, (**d**) AuMP@4-MBA@Cu^2+^@CN.

The elemental composition of the obtained AuMPs was directly analyzed through energy-dispersive X-ray (EDX) elemental mapping, as illustrated in Figure 3. The mapping reveals that gold (Au), indicated by the yellow color, predominates. Additionally, carbon (C) in green and nitrogen (N) in orange, attributed to poly(N-(3-Amidino)-Aniline) (PNAAN) and polyvinylpyrrolidone (PVP), respectively, are widely distributed across the AuMPs (Figure 3a). This distribution indicates that the AuMPs are coated with layers of PNAAN and PVP. After modifying the AuMPs with 4-MBA, a significant presence of sulfur (S), marked in red, is observed across the AuMPs. This phenomenon results from the self-assembly of 4-MBA on the AuMPs, facilitated by the formation of robust gold-sulfur (Au-S) bonds (Figure 3c). The EDX analysis of AuMP@4-MBA@Cu^2+^@CN (Figure 3d) reveals copper (Cu) in purple firmly attached to the AuMPs, owing to effective carboxylate-Cu^2+^ chelation interactions on the AuMPs’ surface. In stark contrast, AuMPs lacking 4-MBA show no significant attachment of free Cu^2+^ after co-incubation with CuCl_2_ solution, as depicted in Figure 3b. This distinct difference highlights the selective binding of Cu^2+^ exclusively to 4-MBA-modified AuMPs, underscoring the critical role of 4-MBA in mediating this interaction.

### 2.4. Optimization of Experimental Conditions

**Figure 4:**
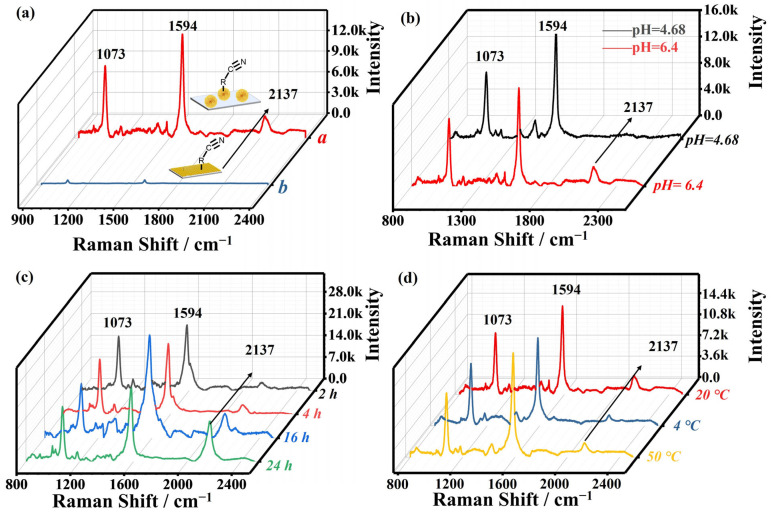
(**a**) The comparison of Raman spectra of AuMP@4-MBA Cu^2+^@CN (line a) and Raman chip@4-MBA Cu^2+^@CN (line b), acquisition time 2 s. (**b**) The Raman spectrum of two pH conditions: CuCl_2_ in PBS (pH = 4.68, black line) and CuCl_2_ in DI (pH = 6.40, red line). (**c**) The Raman spectrum of four reaction time conditions: 2 h, 4 h, 16 h, and 24 h, respectively. (**d**) The Raman spectrum of three temperature conditions: room temperature (20 °C, red line), low temperature (4 °C, blue line), and high temperature (50 °C, yellow line).

To evaluate the SERS enhancement and response of individual AuMPs, a commercial Raman chip procured from Xiamen Spectroscopy Scientific Instruments Co., Ltd. (Xiamen, China), was used for comparison. Both the SERS probe and the Raman chip were functionalized with 4-MBA and applied for Cu^2+^ detection. Figure 4a shows the significant enhancement provided by the SERS probe (acquisition time: 2 s), where the peaks at 1073 cm^−1^ and 2137 cm^−1^ on AuMPs are 23.32-fold and 106.88-fold higher, respectively, compared to those on the commercial Raman chip. SERS EF were calculated as 1.39 × 10^6^ for AuMPs and 1.30 × 10^4^ for the Raman chips. This experiment demonstrates the superior signal-amplification performance of AuMPs, validating their outstanding effectiveness in enhancing SERS signals.

To better explore the Cu^2+^ detection performance, three key parameters were optimized: the pH of the Cu^2+^ solution, the incubation time, and the reaction temperature. The determination of Cu^2+^ concentration is significantly influenced by the solution pH because of the electrostatic interaction underlying the binding of Cu^2+^ with 4-MBA, which is negatively charged. Additionally, Cu^2+^ tends to precipitate in basic solutions. Considering these factors, two critical pH values, 4.68 and 6.40, were selected for the pH-control experiment. Notably, the benzene ring structure in 4-MBA provides a characteristic and stable -CH stretching vibration peak at 1073 cm^−1^ [50], suitable for use as an internal standard peak. This approach has been successfully applied in our prior studies [21,23,29,30] and other reports [50,51,52]. Thus, in this study, the intensity ratios of the peak at 2137 cm^−1^ relative to those at 1073 cm^−1^ can effectively mitigate errors associated with absolute intensity measurements, thereby enhancing the accuracy and reliability of the data interpretation. Each spectrum underwent baseline removal and data smoothing, and the intensity of the distinctive peak was normalized as an internal standard.

In Figure 4b, it is clear that no peak appears in the silent region when the pH is 4.68 (black line). However, a prominent Raman peak at 2137 cm^−1^ is observed at pH 6.40 (red line), I_2137_/I_1073_ = 0.286. This phenomenon is attributed to the complete dissociation of -COOH on the AuMP surface and the subsequent interaction among carboxylate groups, Cu^2+^ and [Fe(CN)_6_]^3−^. When examining the impact of reaction time on Cu^2+^ detection, different incubation periods were tested. The corresponding SERS spectra (Figure 4c) showed a gradual increase in the intensity of the 2137 cm^−1^ peak over time, with the I_2137_/I_1073_ ratios measured as 0.147, 0.173, 0.441, and 0.741 at reaction times of 2 h, 4 h, 16 h, and 24 h, respectively. This enhancement results from the increasing number of effective copper-carboxylate coordinations. Notably, the intensity of the 2137 cm^−1^ peak after 24 h is 5.03 times higher than that after 2 h. To optimize the detection efficiency, a reaction time of 4 h was selected as the optimal condition because its intensity was significantly higher than that of 2 h. Figure 4d indicates the Raman results for the optimization of the reaction temperature throughout the incubation of Cu^2+^. The intensity of 20 °C is 2.15 times and 1.30 times higher than that of 4 °C and 50 °C, the I_2137_/I_1073_ ratios measured as 0.286, 0.173, and 0.220 at reaction temperature of 20 °C, 4 °C, and 50 °C, respectively. To obtain the best detection results, we finally chose a pH of 6.40 in deionized (DI) water, a reaction time of 4 h, and a reaction temperature of 20 °C as the optimal conditions.

### 2.5. Algorithm Based Data Processing

**Figure 5:**
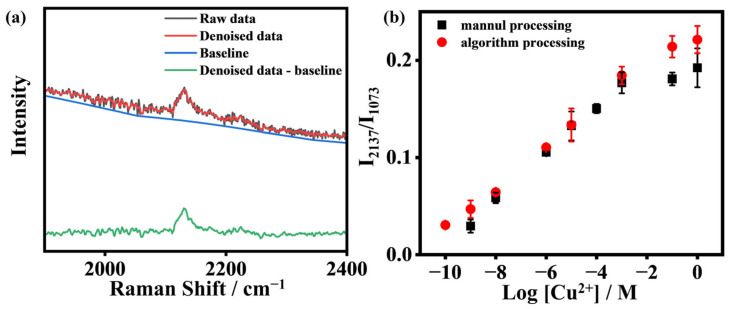
(**a**) The spectrum in the silent region at a concentration of 1 μM after noise reduction and baseline correction. (**b**) The I_2137_/I_1073_ ratio of the concentration of CuCl_2_ from 1 nM to 1 M of manual processing and 100 pM to 1 M of algorithm processing.

The Raman spectra processing algorithm consists of two steps designed to enhance precision and reliability. First, it focuses on optimizing the signal-to-noise ratio of the original data. The algorithm applies the Savitzky–Golay (SG) filter, which uses advanced signal extraction techniques to separate relevant signals from noise, carefully maintaining the intensity and peak characteristics of positive characteristic peaks. Second, for accurate baseline correction, the algorithm applies the adaptive iteratively reweighted penalized least squares (airPLS) algorithm, developed by Zhang et al. [53]. In comparison with conventional methods, herein called manual processing, in which noise reduction and baseline correction are performed subjectively by operators, algorithmic processing utilizes standardized, automated workflows (e.g., Savitzky–Golay (SG) filtering and adaptive iteratively reweighted penalized least squares (airPLS)) to objectively extract signals and correct baselines, thereby ensuring reproducibility and minimizing human-induced biases. As shown in Figure 5a, we used the Raman signal of 1 μM Cu^2+^ for demonstration. The gray line represents the original data, and the green line represents the data processed by the algorithm. The algorithm’s integration of advanced signal extraction and robust baseline correction techniques demonstrates its effectiveness in processing complex spectral data, making it a valuable asset for advanced analytical applications. Furthermore, Figure 5b presents a plot of I_2137_/I_1073_. Notably, the algorithm-processed ratio points (red points) are significantly higher than the manually processed ones (black points). Simultaneously, the detection range is broader. These results underscore the precision and sensitivity achieved through our algorithm-optimized data. By introducing the algorithm, we substantially minimize instrumental errors and human inaccuracies associated with manual processing. Our optimized algorithm not only refines and enhances sensitivity but also effectively eliminates errors introduced by both instruments and human intervention.

### 2.6. SERS Detection of Cu^2+^

**Figure 6:**
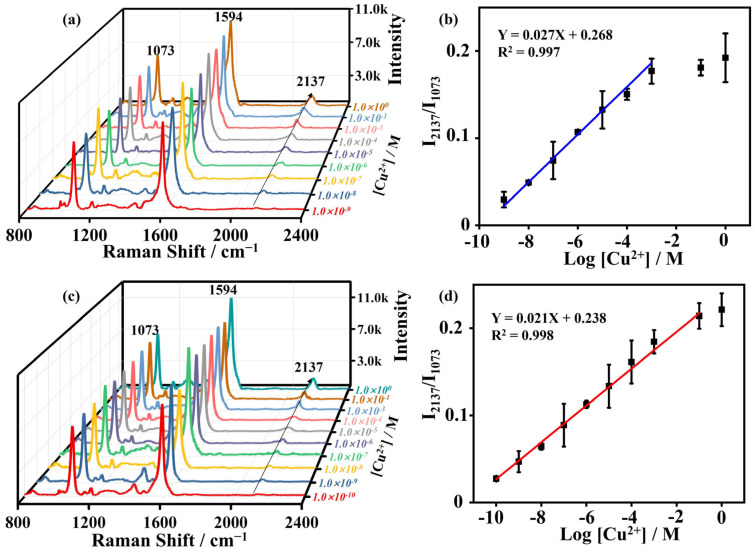
(**a**) The Raman spectrum of AuMP@4-MBA that reacted with different CuCl_2_ concentrations of: 1 nM, 10 nM, 1 μM, 10 μM, 100 μM, 1 mM, 100 mM, and 1 M based on manual baseline correction, respectively. (**b**) Linear equation and linear range fitted after manual processing. (**c**) The Raman spectrum of AuMP@4-MBA that reacted with different CuCl_2_ concentration of: 100 pM, 1 nM, 10 nM, 1 μM, 10 μM, 100 μM, 1 mM, 100 mM, and 1 M based on algorithm baseline correction, respectively. (**d**) Linear equation and linear range fitted after algorithm processing.

Although the Raman fingerprint in the silent range exhibits a relatively high signal-to-noise ratio, the baseline shows deviations from flatness, which may significantly affect the sensing capability. To further enhance the sensitivity and accuracy of this detection scheme, we introduced an algorithm to analyze and calculate the spectra through signal-to-noise ratio optimization and baseline correction. Figure 6a presents the results of the concentration plot for Cu^2+^ detection after manual processing. The experimental results ranged from 1 nM to 1 M, as the concentration increased, the 2137 cm^−1^ peak intensity also increased. In Figure 6c, Cu^2+^ detection concentrations ranged from 100 pM to 1 M after algorithm processing. As the concentration increases, the 2137 cm^−1^ peak rises, displaying a linear relationship with the intensity of the characteristic peak at 1073 cm^−1^. Moreover, this algorithm can be further tailored for optimizing effects, enabling automatic detection of Cu^2+^ concentrations in solutions. It also holds promise for similar optimizations and automated detection in the analysis of various other markers in solution-based assays. The manually processed equation was determined as Y = 0.027X + 0.268 with an R^2^ value of 0.997, operating within the linear range of 1 × 10^−9^ M to 1 × 10^−3^ M (Figure 6b, the corresponding limit of detection (LOD) is 1 nM. In contrast, the algorithm-processed equation yielded values of Y = 0.021X + 0.238 and an R^2^ value of 0.998, spanning a linear range from 1 × 10^−10^ M to 1 × 10^−1^ M (Figure 6d). The LOD was 0.1 nM, which was lower than that of manually processed SERS data. Furthermore, this SERS sensor exhibited superior sensitivity compared to reported methods for Cu^2+^ determination, including colorimetric, fluorometric, HPLC, MS, and electrochemical approaches. Notably, its sensitivity also surpassed traditional SERS sensors (Table 1). All spectra were standardized using the -CH stretching vibration of the benzene ring of 4-MBA at 1074 cm^−1^. Each spectrum underwent baseline removal and data smoothing, and the intensity of the distinctive peak was normalized as an internal standard.

### 2.7. Specificity of SERS Sensing

**Figure 7:**
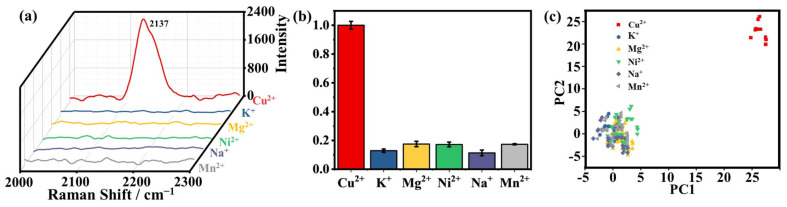
(**a**) The Raman spectra of Ni^2+^, Na^+^, Mn^2+^, K^+^, Mg^2+^, and Cu^2+^ in the range of 2000–2300 cm^−1^ in the silent region. (**b**) Taking the I_2137_/I_1073_ ratio of 10 mM Cu^2+^ as “1.0” and calculating the relative ratio of other ions result, Ni^2+^, Na^+^, Mn^2+^, K^+^, and Mg^2+^. (**c**) PCA scores plots of the different ions and Cu^2+^.

To validate the specificity of 4-MBA and [Fe(CN)_6_]^3−^ in detecting Cu^2+^, interference experiments were carefully carried out. A set of common substances, including Ni^2+^, Na^+^, Mn^2+^, K^+^, and Mg^2+^, each at a concentration of 100 mM, was chosen for the experiments. These substances were made to undergo an oscillatory reaction with the SERS probe for 4h. After that, they were thoroughly washed and then incubated with K_3_[Fe(CN)_6_] prior to Raman characterization. Figure 7a,b shows the results of the ion experiment, presenting the relative intensities compared to 10 mM Cu^2+^ (set as “1.0”). The relative ratios of Ni^2+^, Na^+^, Mn^2+^, K^+^, and Mg^2+^ were determined to be 0.16, 0.14, 0.13, 0.21, and 0.18, respectively. This comparison emphasizes the selectivity of the SERS probe for Cu^2+^ in the presence of various interfering ions. Prior to principal component analysis (PCA), the SERS data went through a two-step optimization process. First, the data were smoothed using an SG filter, and then the airPLS technique was employed to adjust the background signal. During the baseline-smoothing process, the polynomial order was set at 1, and the lambda value for parameter adjustment was fixed at 100. These careful pre-processing steps were crucial to ensure that the SERS data were properly prepared for PCA analysis. Figure 7c demonstrates the successful differentiation of Cu^2+^ (represented by red markers) from other interfering ions at various positions. Significantly, the positions representing Cu^2+^ do not overlap with those of interfering ions, indicating the effectiveness of the PCA in differentiating Cu^2+^ from the interference of other ions.

### 2.8. Detection in Serum Samples

The detection of Cu^2+^ in human serum was performed to evaluate the detection reliability in real samples. First, the human serum was diluted ten-fold with DI water. Subsequently, known concentrations of Cu^2+^ and the SERS probe were added. After additional incubation, the SERS signals were measured. The results, presented in Table 2, detail the recovery rates and relative standard deviations (RSD) of Cu^2+^. At concentrations of 10 μM, 1 μM, and 0.1 μM, the mean recoveries were found to be 97.63%, 101.34%, and 104.41%, respectively. The corresponding RSDs were 8.90%, 7.54%, and 8.27%. These findings highlight the probe’s high sensitivity and excellent reproducibility, suggesting its substantial potential for clinical diagnostics and studies related to disease mechanisms.

## 3. Materials and Methods

### 3.1. Materials and Instruments

4-Mercaptobenzoic acid (4-MBA), polyvinylpyrrolidone (PVP), *N*-methyl-2-pyrrolidone (NMP), chloroauric acid (HAuCl_4_), copper (II) chloride dihydrate (CuCl_2_·2H_2_O), potassium ferricyanide (K_3_[Fe(CN)_6_]), anhydrous ethanol, and hydrochloric acid were purchased from Sigma. *N*-(3-amidine)-aniline (NAAN) was synthesized according to the reported method [64]. All chemicals were analytical grade and used as received. Deionized water (DI, >18.0 MΩ cm) was purified by the Millipore Milli-Q gradient system (Burlington, MA, USA).

Scanning electron microscope (SEM) images of the morphology and energy dispersive X-ray (EDX) mapping images of Au microparticles (AuMPs) were recorded by field emission scanning electron microscopy (FESEM) (Ultra-55, ZEISS, Jena, Germany). Raman spectra were measured on a Laser Confocal MicroRaman Spectrometer (LabRAM HR Evolution, HORIBA Jobin Yvon S.A.S, Tokyo, Japan) equipped with a 633 nm laser excitation source.

### 3.2. Preparation of SERS Substrate

AuMPs were synthesized using NAAN as a reducing agent and PVP as a co-capping agent according to the reported method [47]. Briefly, 8 μL of a 10% (wt%) HauCl_4_ solution and 25 μL of a 32 mg/mL PVP solution were added to 959 μL of a 1 mM HCl aqueous solution. The mixture was incubated at 4 °C for 2 h. Subsequently, 8 μL of a 100 mg/mL NAAN solution was added, and the resulting mixture was incubated for 24 h at 4 °C. The resultant solution was then centrifuged at 6000 rpm. The collected pellets were washed with NMP and DI water to remove organic reactants and impurities.

### 3.3. Procedure for SERS Detection

10 μL of the SERS probe was incubated with 200 μL of 100 mM CuCl_2_ for 4 h at room temperature and washed three times with DI water to remove the free Cu^2+^. Then, the obtained probes were added to 200 μL of 100 mM K_3_[Fe(CN)_6_] for 20 min, and the excess [Fe(CN)_6_]^3−^ was removed by washing. The SERS signals were recorded after 2.5 μL of the final particles were dried on a glass slide. To investigate the sensitivity of this detection method, various concentrations of Cu^2+^ (ranging from 100 pM to 1 M) were used, and the other procedures were the same as those mentioned above. The SERS measurements of the probes were conducted using a Laser Confocal MicroRaman Spectrometer (LabRAM HR Evolution, HORIBA Jobin Yvon S.A.S, Tokyo, Japan). The system was equipped with a 633 nm laser with an incident laser power of 1 mW and a spot size of 2 μm.

To investigate the selectivity of the designed probe, various ions were utilized to perform the detection procedure following the same protocol mentioned above. The ions included Ni^2+^, Na^+^, Mn^2+^, K^+^, and Mg^2+^. The tested concentrations of the above ions were 100 mM each.

### 3.4. Detection of Cu^2+^ in Serum Samples

Then, the anti-interference ability of this method to impurities in complex samples was investigated. For this purpose, the recovery of Cu^2+^ in human serum was detected. The human serum was supplied by The First Affiliated Hospital of Jinan University (Guangzhou, China). The serum was centrifuged, and the collected supernatant was diluted by 10-fold with PBS. Briefly, three equivalent volumes of Cu^2+^ solution were added into serum to obtain different concentrations of 10 μM, 1 μM, and 100 nM.

### 3.5. Algorithm Processing

For the PCA, the SERS data were optimized through a two-step process. First, a SG filter was applied to smooth the data, and then background correction was performed using the airPLS method, the maximum iteration limit was set at 15, the parameter lambda, with a value of 100 and a polynomial order of 1, was used to regulate the level of baseline smoothing. Before PCA analysis, these steps were followed to ensure that the SERS data were adequately pre-processed. The PCA provides a comprehensive exploration of complex molecular interactions captured by SERS spectra. The computation of the covariance matrix enabled the extraction of eigenvalues and corresponding eigenvectors, thus determining the principal components. These components represent directions in the feature space where the data show significant variance.

## 4. Conclusions

To overcome drawbacks such as the weak signal intensity and large background interference in SERS biosensing, a new SERS method was developed by combination of silent region fingerprints with a signal-amplification strategy through the introduction of ferricyanide molecules with many cyano groups by the specific coordination of Cu^2+^ which was adsorbed on the AuMPs surface. There are two main merits in our proposed method. One is that the number of cyano groups on the SERS substrate can generate a strong and stable signal in the SERS silent-region, and such signal intensity is correlated with Cu^2+^ concentration, resulting in a novel SERS method for Cu^2+^ detection with high sensitivity and selectivity; another is that benefiting from per ferricyanide possessing six cyano groups, the established method with the advantage of signal amplification can significantly enhance the sensing sensitivity. Moreover, an algorithm data processing for the optimization of SERS original data was further used to improve the SERS signal reliability. The experiments results demonstrate that the novel SERS sensor for Cu^2+^ detection showed a broad linear range, low limit of detection, and high selectivity, which is promising for quantitative analysis in complicated biological samples. The possibility of reuse of SERS substrates is attractive, and we will continue research on the reuse of SERS substrates. In summary, our work paves the way for improving SERS sensing capability through the silent-range fingerprint and signal amplification strategy, which reveals SERS as an effective tool for trace detection.

## Figures and Tables

**Table 1:** Cu^2+^ Detection by SERS and other methods.

Method	Linear Range (M)	LOD (M)	Ref
Fluorescence	0–1.0 × 10^−5^	5.0 × 10^−10^	[54]
Colorimetric	5.0 × 10^−7^–1.0 × 10^−5^	2.5 × 10^−7^	[55]
Colorimetric	1.6 × 10^−8^–1.3 × 10^−7^	5.0 × 10^−10^	[56]
HPLC	1.0 × 10^−8^–5.0 × 10^−7^	1.9 × 10^−9^	[57]
MS	1.0 × 10^−8^–1.0 × 10^−5^	3.5 × 10^−9^	[58]
Electrochemistry	1.0 × 10^−8^–1.0 × 10^−4^	5.0 × 10^−9^	[59]
SERS	5 × 10^−10^–1.0 × 10^−6^	1.8 × 10^−10^	[60]
SERS	5.0 × 10^−8^–5 × 10^−4^	3.5 × 10^−8^	[61]
SERS	2.5 × 10^−7^–2.5 × 10^−4^	8.0 × 10^−9^	[62]
SERS	3.0 × 10^−5^–9 × 10^−5^	2.2 × 10^−6^	[63]
SERS	1.0 × 10^−10^–1.0 × 10^−1^	1.0 × 10^−10^	This work

**Table 2:** The physiology experiment of Cu^2+^ in serum.

Sample	Predicted Conc. (μmol/L)	Detected Conc. (μmol/L)	Recovery (%)	RSD (%)
1	10.00	9.76 ± 0.89	97.63	8.90
2	1.00	1.01 ± 0.08	101.34	7.54
3	0.10	0.10 ± 0.00	104.41	8.27

## Data Availability

Data is contained within the article.
